# Electrical and Structural Properties of Si_1−*x*_Ge*_x_* Nanowires Prepared from a Single-Source Precursor

**DOI:** 10.3390/nano13040627

**Published:** 2023-02-04

**Authors:** Raphael Behrle, Vanessa Krause, Michael S. Seifner, Benedikt Köstler, Kimberly A. Dick, Matthias Wagner, Masiar Sistani, Sven Barth

**Affiliations:** 1 Institute of Solid State Electronics, TU Wien, Gußhausstraße 25-25a, 1040 Vienna, Austria; 2 Institute of Physics, Goethe University Frankfurt, Max-von-Laue-Str. 1, 60438 Frankfurt, Germany; 3 Centre for Analysis and Synthesis, Lund University, P.O. Box 124, 22100 Lund, Sweden; 4 Institute for Inorganic and Analytical Chemistry, Goethe University Frankfurt, Max-von-Laue-Str. 7, 60438 Frankfurt, Germany

**Keywords:** nanowires, silicon, germanium, alloy, CVD, field-effect transistors

## Abstract

Si_1−*x*_Ge*_x_* nanowires (NWs) were prepared by gold-supported chemical vapor deposition (CVD) using a single-source precursor with preformed Si–Ge bonds. Besides the tamed reactivity of the precursor, the approach reduces the process parameters associated with the control of decomposition characteristics and the dosing of individual precursors. The group IV alloy NWs are single crystalline with a constant diameter along their axis. During the wire growth by low pressure CVD, an Au-containing surface layer on the NWs forms by surface diffusion from the substrate, which can be removed by a combination of oxidation and etching. The electrical properties of the Si_1−*x*_Ge*_x_*/Au core-shell NWs are compared to the Si_1−*x*_Ge*_x_* NWs after Au removal. Core–shell NWs show signatures of metal-like behavior, while the purely semiconducting NWs reveal typical signatures of intrinsic Si_1−*x*_Ge*_x_*. The synthesized materials should be of high interest for applications in nano- and quantum-electronics.

## 1. Introduction

Semiconductor nanowires (NWs) play a major role in intriguing technological applications in the field of high-performance nanoelectronics [1], photovoltaics [2], photonics [3], thermoelectrics [4,5], chemical energy storage [6], and biomedicine [7]. Different aspects of semiconductor NW research have been reviewed and the fundamental growth, structure, and property relations have been described [8,9,10,11,12,13,14]. Among this wide field, group IV materials are of specific interest as these materials could be easily integrated into current device processing approaches.

Today, Si_1−*x*_Ge*_x_* is already a key material in modern high-speed bipolar transistors. Most notably, Si_1−*x*_Ge*_x_* and Ge have been identified as promising channel materials for field-effect transistors (FETs) to enable higher drive currents, a reduction in dynamic power consumption, and enhanced switching speeds compared with conventional Si devices [15]. The group IV substitutional solid solution Si_1−*x*_Ge*_x_* is an alloy with complete solubility over the whole composition range [16]. Si_1−*x*_Ge*_x_* nanostructures are extensively used in a large portfolio of applications including advanced transistors, quantum devices, photodetectors, electro-optical modulators, photovoltaics, microelectromechanical systems (MEMS), and thermoelectric generators [17,18,19,20,21,22,23]. Moreover, Si_1−*x*_Ge*_x_* interlayers can be used to control strain and defect densities in Si and Ge layers for electrical applications in CMOS device architectures [24,25,26]. In addition, the first significant observations of band edge photoluminescence were also based on fully strained Si_1−*x*_Ge*_x_* [27,28]. The electrical properties of the Si_1−*x*_Ge_*x*_ substitutional solid solution with cubic crystal phase have been summarized [29], but new developments can benefit from molecular precursors providing pre-formed Si-Ge building blocks.

The controlled synthesis of thin layers and nanostructures of Si_1−*x*_Ge*_x_* is typically based on molecular beam epitaxy using the elements as sources [30,31,32] and mixtures of SiH_4_/GeH_4_ as precursors in chemical vapor deposition [33,34]. Similarly, Si_1−*x*_Ge*_x_* NW growth has been described by metal-supported CVD using individual Ge and Si precursors by various groups [35,36,37].

The single-source precursor approach was successfully demonstrated for different binary materials in the past [38,39,40,41]. More specifically, previously described single-source precursors for Si_1−*x*_Ge*_x_* material carrying exclusively hydride ligands are pyrophoric and require rigorous safety measures similar to the individual SiH_4_ and GeH_4_ sources [42,43], which makes the approach described here using a less reactive precursor intriguing. For the thermal conversion of precursors to Si_1−*x*_Ge*_x_*, it should be noted that Si–C-containing silanes typically lead to silicon carbide [44,45,46,47], while Ge–C can be cleaved even at very moderate temperatures, yielding pure Ge material [48,49,50]. Applications based on non-pyrophoric single-source precursors for Si_1−*x*_Ge*_x_* synthesis combine easier handling with a simplified parameter set to control the materials’ synthesis, representing a viable alternative to the conventional approaches.

Here, we report on the Au-assisted CVD synthesis of NWs using (H_3_Si)_2_Ge(*n*Bu)_2_ precursor and their structural as well as chemical characterization by μ-Raman spectroscopy, energy dispersive X-ray spectroscopy (EDX), scanning electron microscopy (SEM), and transmission electron microscopy (TEM). The electrical properties were investigated in two- and four-probe geometry and reveal differences in the behavior between Si_1−*x*_Ge*_x_*/Au core–shell NWs and Si_1−*x*_Ge*_x_* NWs.

## 2. Materials and Methods

### CVD Process and Thin Film Characterization

CVD was carried out in a home-built cold-wall reactor using high-frequency heating of a graphite or steel susceptor for indirect heating of sapphire (0001) (Crystal GmbH, Berlin, Germany). Prior to use, the substrates are coated with a ~5 nm Au film by sputtering. The substrates are attached to the susceptor by silver paste to ensure efficient thermal contact. Substrate temperatures were limited to *T*_S_ = 750–783 K. The precursor was introduced into the reactor through a glass flange applying a dynamic vacuum (~10^−6^ mbar) while keeping the precursor temperatures in the range of 253–258 K using a cooling bath based on chilled isopropyl alcohol as a coolant. Typically, 40–80 mg of the (H_3_Si)_2_Ge(*n*Bu)_2_ precursor was used as source for the CVD experiments and the growth was carried out for 60–90 min. Growth experiments with similar parameters were verified at least three times. Typically, higher substrate temperatures also result in higher density of NWs, but differ in the microstructure as described below. The detailed description of the precursor synthesis has been published recently [51]. A similar CVD setup has been described in the literature for the growth of thin films and nanostructures using molecular sources [52,53].

The Au removal was carried out by post-growth oxidation at 1173 K for 20 min under 50 sccm oxygen flow. The oxide was removed by 60 s etching using buffered HF, which is an ammonium fluoride etching mixture solution (Sigma-Aldrich, St. Louis, MO, USA). Finally, the Au was removed by 60 s etching using commercial KI/I_2_ solution purchased from Sigma-Aldrich. The NWs were washed twice with deionized water and once with isopropyl alcohol. Electron beam lithography was used to define the electrodes and Al was sputtered as electrode material using similar steps as described in the literature [54] and an additional HF dip before Al deposition.

A JEOL JEM-3000F equipped with a Schottky field-emission electron source operating at 300 kV was used to acquire TEM images, HAADF-STEM images, STEM-EDX line scans, and STEM-EDS elemental maps. TEM images were recorded with a charge-coupled device (CCD) camera (GATAN Orius camera). An Oxford Instruments INCA system and an 80 mm^2^ silicon drift detector (SDD) were used to perform EDX analyses. The data were acquired and processed with DigitalMicrograph from Gatan (Version 2.31.734.0/DigitalMicrograph™ 3.9.3 for GMS 1.4.3) and INCA from Oxford Instruments Nanotechnology Tools Ltd. (Version 4.15). 

The μ-Raman measurements were performed on a WITec Alpha300 Raman system with a frequency-doubled Nd:YAG laser (λ = 532 nm) in a backscattering geometry. The power of the incident laser was adjusted to 100 μW to prevent heated up of the NWs. The laser was focused through an achromatic Nikon EPI EPlan 100× objective (NA = 0.9, WD = 0.23 mm), enabling a diffraction limited spot size of ~720 nm. The integration time was set to 60 s.

The electrical measurements were conducted at room temperature and ambient conditions using a HP 4156B semiconductor analyzer and a probe station placed in a shielded dark box to exclude the influence of ambient light and electromagnetic fields.

## 3. Results

**Figure 1:**
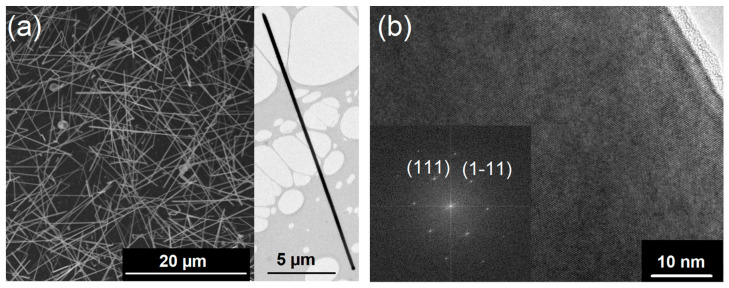
(**a**) The SEM image of Si_1−*x*_Ge*_x_* NWs grown at 773 K on Au-coated sapphire substrates shows a high number of very long NWs (>20 μm). The TEM image in the inset illustrates the low tendency for tapering of the NWs under these growth conditions. (**b**) A high-resolution TEM image shows the single crystalline nature of the NWs and the corresponding FFT verifies the growth of the Si_1−*x*_Ge*_x_* crystal along the 〈111〉 axis.

Low-pressure CVD (LPCVD) without a carrier gas was used to grow Si_1−*x*_Ge*_x_* NWs on Au-coated sapphire substrates. The recently described single-source precursor (H_3_Si)_2_Ge(*n*Bu)_2_ containing Si and Ge in one molecule was used for the NW synthesis [51]. This molecular source reduces parameters for the precursor delivery when compared with individual precursors and, at the same time, its reduced reactivity allows for more convenient handling without elaborate safety measures. For NW growth, the liquid precursor (H_3_Si)_2_Ge(*n*Bu)_2_ was kept at 250–255 K during the LPCVD owing to its high vapor pressure. Figure 1a illustrates the efficient formation of Si_1−*x*_Ge*_x_* NWs on Au-coated sapphire substrates. The NW density, very low tapering tendency, and their length are ideal for following electrical characterization. The expected Au seed is always observed at the tip of each NW, which is a signature of the NW growth according to the vapor–liquid–solid (VLS) mechanism, as described first for the growth of Si NWs [55] and investigated in detail over the last decades for various systems [8,56,57].

High-resolution transmission electron microscopy (HR-TEM) images, as shown in Figure 1b, reveal the formation of Si_1−*x*_Ge*_x_* single crystals. Moreover, the fast Fourier transform (FFT) of the HR-TEM image in Figure 1b shows the crystal growth along the 〈111〉-axis, as expected for group IV NWs of these diameters [58]. Typically, a thin amorphous shell is observed, which is caused by a simultaneous layer formation of amorphous Si_1−*x*_Ge*_x_* and the crystalline NW. However, the metal-supported NW growth is much more efficient than an amorphous layer formation at such low growth temperatures of 773 K as described herein.

**Figure 2:**
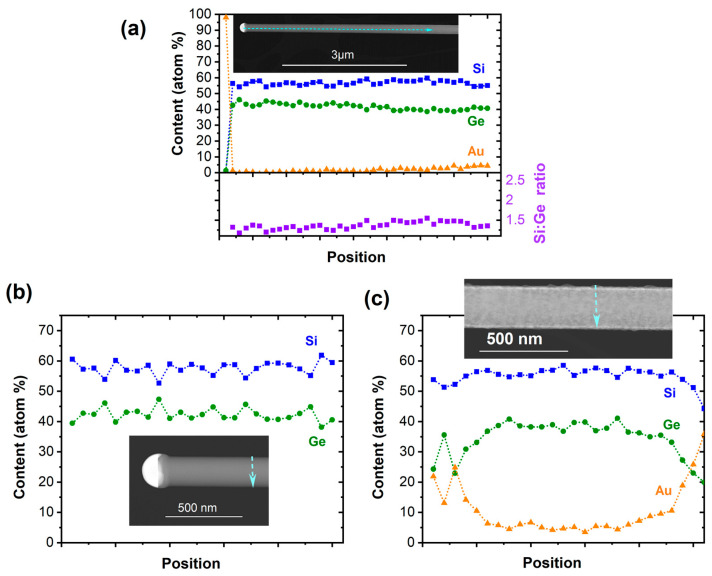
(**a**) Elemental distribution of Si, Ge, and Au as well as Si/Ge ratio along the NWs’ axis. The EDX line scan perpendicular to the NW growth axis in (**b**) shows the group IV elemental distribution of the NW in proximity to the growth seed where no Au has been recorded. (**c**) The formation of a Si_1−*x*_Ge*_x_*/Au core–shell NW towards the NW base is illustrated in the EDX line scan perpendicular to the NW growth axis.

The Si_1−*x*_Ge*_x_* NWs composition was determined by energy dispersive X-ray (EDX) spectroscopy. Figure 2a reveals that the NWs contain a ~1.5–1.3:1 ratio of Si/Ge instead of the predefined ratio of 2:1 in the (H_3_Si)_2_Ge(*n*Bu) precursor. This suggests a variation in the decomposition path in the Au-assisted growth, as the Si/Ge ratio is retained in the CVD growth of amorphous layers from the same precursor [51]. Scrambling reactions on the Au surface leading to volatile Si-containing fragments could rationalize the loss of Si. The slow film formation from the same precursor at 773 K in absence of Au causes a thin, negligible overlayer of amorphous Si_2_Ge. In proximity of the growth seed, no Au has been reliably recorded, but the Au content on the surface of NWs increases over the length of several micrometers, as illustrated in Figure 2a.

The cross-sectional EDX line scan in proximity to the growth seed shows no Au surface coverage and the formation of a homogeneous material. In agreement with the observation of a significant Au content along the NW, a cross-sectional EDX line scan closer to the NW base illustrates the surface accumulation of Au and the formation of a Si_1−*x*_Ge*_x_*/Au core–shell structure (Figure 2c). The minor change in the NWs’ diameter over the length of several micrometers suggests that the Au diffuses along the NWs from the substrate surface that acts as the reservoir, while the Au content along the wire appears to be constant, as illustrated in Figure S1 (Supplementary Material). Moreover, a loss of Au from the growth seed would lead to a significant tapering of the NWs, which is not observed herein. The Au-containing shell is accompanied by slight roughening of the surface, as illustrated in Figure S1. The roughness of the surface and comparably low number of NWs originating from a 5 nm Au film suggest only limited activation of Au growth seeds in the initial stage. As a consequence, the Au is covered with a thin film, preventing further nucleation of NWs. The density of NWs is higher with the increasing growth temperature, but at the same time, an amorphous Si_1−*x*_Ge*_x_*-containing shell forms as a result of the competing thin film growth, as discussed below. It should be noted that the Z-contrast within the tips illustrated in the inset of Figure 2b is caused by the high solubility of the group IV semiconductors in Au at a growth temperature of 773 K and the associated phase separation and further growth of the semiconductor segment upon cooling.

As the CVD is carried out at the lowest decomposition temperatures for (H_3_Si)_2_Ge(*n*Bu) fragmentation, and thus in the reaction limited regime, a small increase in the substrate temperature will favor the simultaneous formation of an amorphous Si_2_GeC_0.1_ layer (Figure S2, Supplementary Material). The consequence is tapering due to the formation of an amorphous Si_2_GeC_0.1_ layer, which forms in this temperature regime in absence of Au as a growth promoter [51]. The difference in composition is illustrated in Figure S3 of the Supporting Information with the amorphous layer containing a higher Si content than the inner core. Even though the Au diffusion on the NWs is prevented, the formation of the Si_2_GeC_0.1_-based layer would require etching of the amorphous layer while keeping the crystalline core intact, which requires elaborate selective etching. Therefore, the focus is on the Si_1−*x*_Ge*_x_*/Au core–shell in the following investigations.

Removal of the Au from the Si_1−*x*_Ge*_x_* NWs’ surface using commercial KI/I_2_ etchant was unsuccessful, which is probably due to partial alloying of Au at the growth temperatures and the formation of a protecting semiconductor-based surface termination [59,60]. Therefore, a harsh thermal dry-oxidation at 1173 K was carried out to oxidize the potential semiconductor shell and subsequently remove the Si_1−*x*_Ge*_x_*O_2−*y*_ by buffered HF. This enabled the Au etching using a commercial KI/I_2_ solution. The number of longer NWs is lower after the procedure and more fragments are encountered owing to the Au diffusion and agglomeration, leading to segmented NWs as intermediates [61]. However, for Si_1−*x*_Ge*_x_*/Au NWs exhibiting mostly surface agglomeration of Au, intact NWs with a rough surface can be obtained that allow for the study of the Si_1−*x*_Ge*_x_* NW cores’ electronic properties after the Au removal. Figure S4 (Supplementary Material) shows a TEM image illustrating the rough surface of the obtained Si_1−*x*_Ge*_x_* NWs. However, it should be noted that the overall composition of the NW core after this treatment is close to SiGe, showing a preferred etching of Si within the structures. When a Si_1−*x*_Ge*_x_* alloy is oxidized, the oxidation potential of Si is sufficiently greater than that of Ge, such that Si is preferentially oxidized and Ge is rejected, which results in a pileup of epitaxial, single-crystal SiGe at the SiO_2_/Si_1−*x*_Ge*_x_* interface [62,63,64]. Therefore, a Ge enrichment is observed in the Si_1−*x*_Ge*_x_* NWs presented in this study. A low concentration of Au is not reliably determined by the EDX investigations, but the electronic properties provide a reliable insight into the material’s physical properties, and thus the potential presence of small metal concentrations.

**Figure 3:**
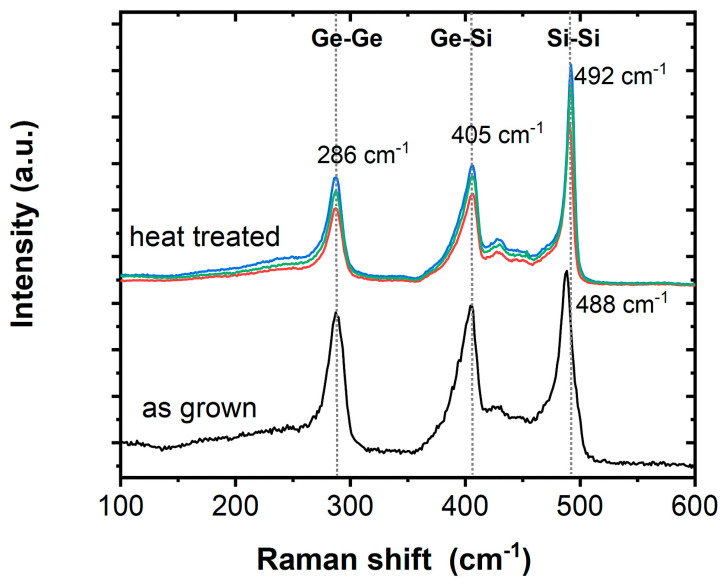
Raman spectra of as-grown Si_1−*x*_Ge*_x_*/Au NWs and Si_1−*x*_Ge*_x_* NWs after high-temperature oxidation, annealing, and etching (abbreviated by heat treated).

In addition to the TEM/EDX analyses, μ-Raman spectroscopy was carried out on Si_1−*x*_Ge*_x_* NWs in order to obtain additional information on the bonding in the substitutional solid solution. Typically, three dominant peaks centered near 300, 400, and 500 cm^−1^ are considered for Ge–Ge, Ge–Si, and Si–Si stretching motions, respectively. The origin of these peaks in Si_1−*x*_Ge*_x_* is well understood [65] and the appearance of the two weak features between 420 and 455 cm^−1^ as well as the shoulder at the low-frequency side of the Si−Si band is regularly observed [66,67]. All Raman spectra herein are shifted vertically for clarity, while the general intensity depends on the number of wires under focus and will vary. Figure 3 shows the typical signals for Si_1−*x*_Ge*_x_* for the as-grown NWs as well as the heat-treated and etched NWs, with the only difference being a slight shift in the Raman peak position associated with the Si−Si mode. The Si−Si band position is the most sensitive to the Ge concentration (*x*) and downshifts rapidly with increasing *x*, while the other two bands are not effected significantly by changes in the composition [67]. The general peak location for the as-grown sample with a peak maximum at 488 cm^−1^ agrees well with literature reports for ~Si_0.6_Ge_0.4_ [67] and the aforementioned Si/Ge ratio for the core material being ~1.5–1.3:1, as determined by EDX. However, the Si/Ge ratio of ~1:1 in the annealed and etched NWs according to EDX does not agree with a small shift in the Raman peak position to 492 cm^−1^, which would be a signature for an increased Si content. Such discrepancies have also been observed in the literature and have been related to either inhomogeneity within batches of NWs or effects of growth temperatures [67].

**Figure 4:**
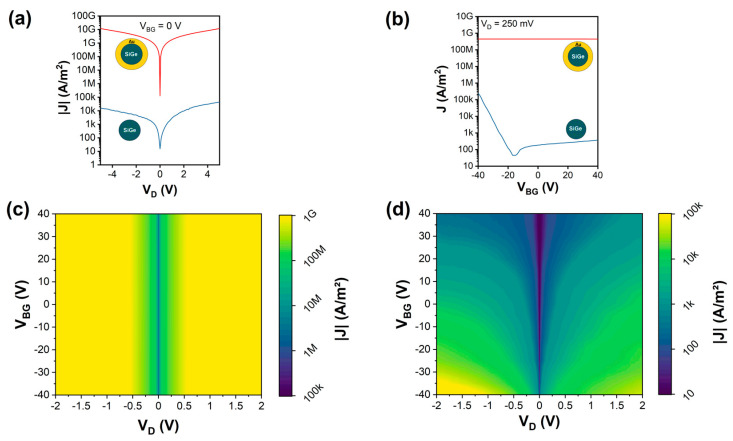
Electrical characterization of as-grown Si_1−*x*_Ge*_x_*/Au NWs and Si_1−*x*_Ge*_x_* NWs: (**a**) current density measurements for V_BG_ = 0 V and sweeping V_D_ from −5 to 5 V; (**b**) transfer characteristic for V_D_ = 250 mV and respective color maps for (**c**) Si_1−*x*_Ge*_x_*/Au NWs and (**d**) Si_1−*x*_Ge*_x_* NWs.

The electrical characteristics of both Si_1−*x*_Ge*_x_*/Au NWs and Si_1−*x*_Ge*_x_* NWs were systematically investigated. Thereto, samples of both NW types were transferred to oxidized, highly p-doped Si substrates. Using a combination of electron-beam lithography, sputter deposition of Al, and lift-off techniques, the NWs were integrated into back-gated Schottky barrier FETs with the Si wafer acting as a common back-gate [68]. In total, for both types of NWs, 19 devices each were fabricated. Figure 4a shows representative I/V measurements of both NW types for grounding the Si substrate (V_BG_ = 0 V). Comparing the I/V characteristics, it is evident that the Si_1−*x*_Ge*_x_*/Au NWs show an approximately six orders of magnitude higher current than the Si_1−*x*_Ge*_x_* NWs. This high conductivity indicates that the main part of the current is flowing through the Au shell of the Si_1−*x*_Ge*_x_*/Au NWs rather than the much more resistive Si_1−*x*_Ge*_x_* core. Further, Figure 4b shows the investigation of the charge carrier modulation capability by the back-gate on both NWs. Applying a bias voltage of V_D_ = 250 mV, a constant transfer characteristic was observed for the NWs with an Au shell, which screens the electric field of the back-gate, resulting in no gate tunability. On the other hand, the NWs without an Au shell showed typical charge carrier modulation capabilities, i.e., a slightly ambipolar characteristic with dominant hole conduction, as expected for an intrinsic Si_1−*x*_Ge*_x_* semiconductor with Al contacts [15,69]. To further map the gate-tunable transport through the proposed NWs, Figure 4c,d show color plots of the recorded current density depending on the applied bias- and gate-voltage. The Si_1−*x*_Ge*_x_*/Au NWs reveal a highly symmetric and gate-independent characteristic. In contrast, the Si_1−*x*_Ge*_x_* NWs show a typical slightly asymmetric I/V characteristic and charge carrier modulation capability by the back-gate voltage [70].

Importantly, the conducted electrical measurements confirm the results from the structural analysis and illustrate that the presented growth grants access to intrinsic Si_1−*x*_Ge*_x_* NWs, which are of high interest for numerous applications in nano- and quantum-electronics [15,23].

## 4. Conclusions

The Au-assisted synthesis of Si_1−*x*_Ge*_x_* NWs was achieved for the first time using a single molecular source as a precursor. In contrast to amorphous layer deposition using (H_3_Si)_2_Ge(*n*Bu)_2_ with retention of the 2:1 Si/Ge ratio, the Au-supported growth leads to Si loss and, typically, a Si/Ge ratio of ~1.5–1.3:1. At higher deposition temperatures, an amorphous layer deposition is observed with the previously reported composition and the overall formation of tapered NWs. Generally, the as-grown NWs are Si_1−*x*_Ge*_x_*/Au core–shell structures and formed by Au surface diffusion from the substrate. The Au shell was successfully removed by an oxidation/etching procedure. The electrical properties reveal typical metallic transfer characteristics for Si_1−*x*_Ge*_x_*/Au NWs, while the Si_1−*x*_Ge*_x_* NWs behave like an intrinsic semiconductor material, as expected. This report demonstrates that highly crystalline Si_1−*x*_Ge*_x_* material with typical semiconductor properties can be prepared by the single-source precursor approach.

## Data Availability

Data presented in this study are available on request from the corresponding author.

## Supplementary Materials

The following supporting information can be downloaded at https://www.mdpi.com/article/10.3390/nano13040627/s1, Figure S1: EDX and TEM images of Si_1−*x*_Ge*_x_*/Au core–shell NWs; Figure S2: TEM images of tapered Si_1−*x*_Ge*_x_* NWs; Figure S3: TEM image and EDX analyses across NWs on thicker and thinner parts of tapered Si_1−*x*_Ge*_x_* NWs; Figure S4: TEM image of heat-treated and etched Si_1−*x*_Ge*_x_* NWs.
