# Supplementary material for: Electrical and Structural Properties of Si1−xGex Nanowires Prepared from a Single-Source Precursor
Source: Nanomaterials (Basel). 2023 Feb 4;13(4):627. doi: 10.3390/nano13040627 (PMC9963966; doi:10.3390/nano13040627)
Supplement: Supplementary file 1 [file nanomaterials-13-00627-s001.zip › nanomaterials-2199423-supplementary.pdf]

Supplementary Materials

Electrical and Structural Properties of $Si_{1-x}Ge_x$ Nanowires Prepared from a Single-Source Precursor

Raphael Behrle [1,†], Vanessa Krause [2,†], Michael S. Seifner [3], Benedikt Köstler [4], Kimberly A. Dick [3], Matthias Wagner [4], Masiar Sistani [1,*], and Sven Barth [2,4,*]

1 Institute of Solid State Electronics, TU Wien, Gußhausstraße 25-25a, 1040 Vienna, Austria
2 Institute of Physics, Goethe University Frankfurt, Max-von-Laue-Str. 1, 60438 Frankfurt, Germany
3 Centre for Analysis and Synthesis, Lund University, Box 124, 22100 Lund, Sweden
4 Institute for Inorganic and Analytical Chemistry, Goethe University Frankfurt, Max-von-Laue-Str. 7, 60438 Frankfurt, Germany

* Correspondence: masiar.sistani@tuwien.ac.at (M.S.); barth@physik.uni-frankfurt.de (S.B.)
† These authors contributed equally to this work.

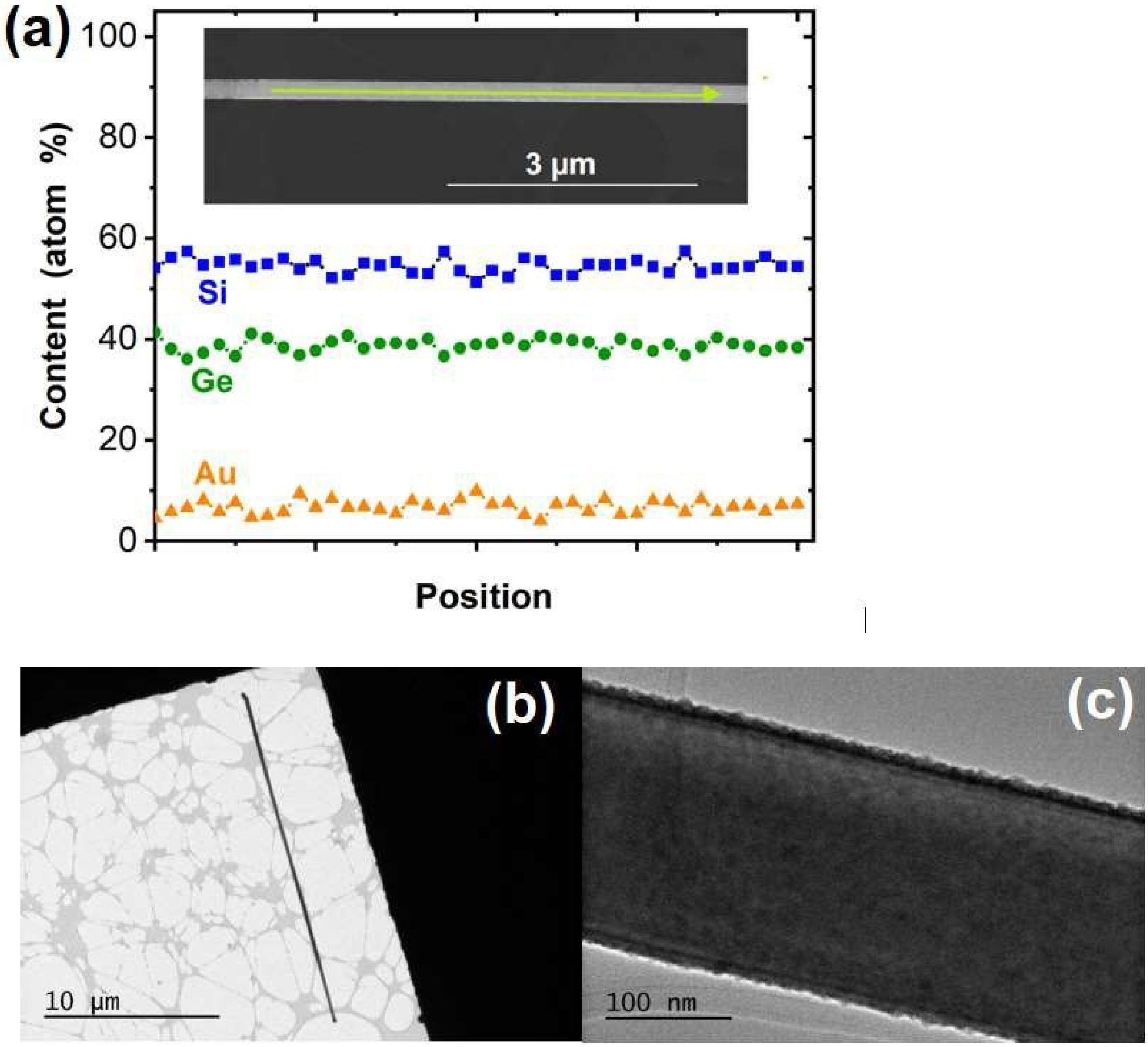

Figure S1. (a) The EDX line scan shows the constant Au coverage along the $Si_{1-x}Ge_x$ farther away from the growth seed. The HAADF image in the inset shows the non-tapered $Si_{1-x}Ge_x$ NW grown at 773 K. (b) An additional TEM image of another untampered $Si_{1-x}Ge_x$ NW grown at 773 K. (c) A higher magnification TEM image reveals the absence of a significant amorphous layer ~15 µm from the growth front. The contrast and rougher surface are caused by the Au-based shell.

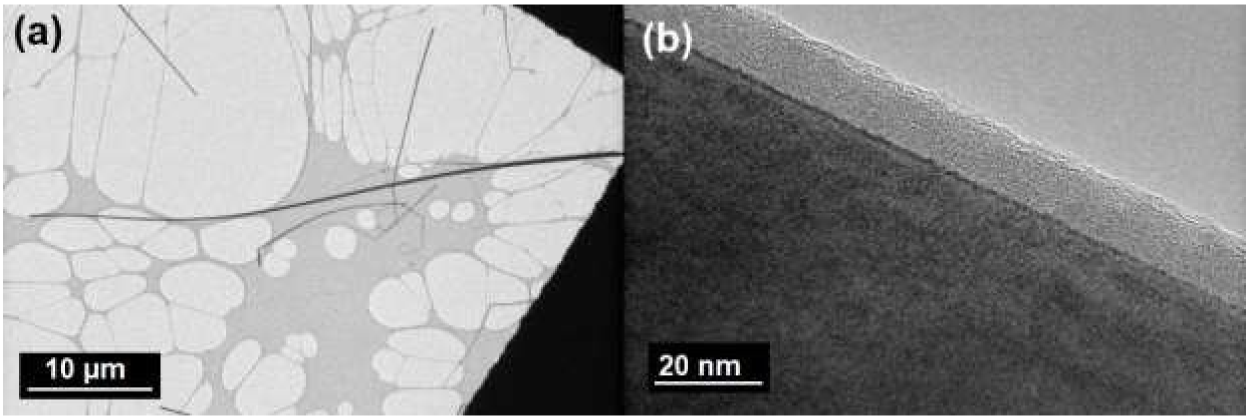

Figure S2. (a) TEM image of tapered $Si_{1-x}Ge_x$ NWs grown at 783 K showing a significant tapering and (b) higher magnification TEM image illustrating the formation of an amorphous layer ~5 µm from the growth front.

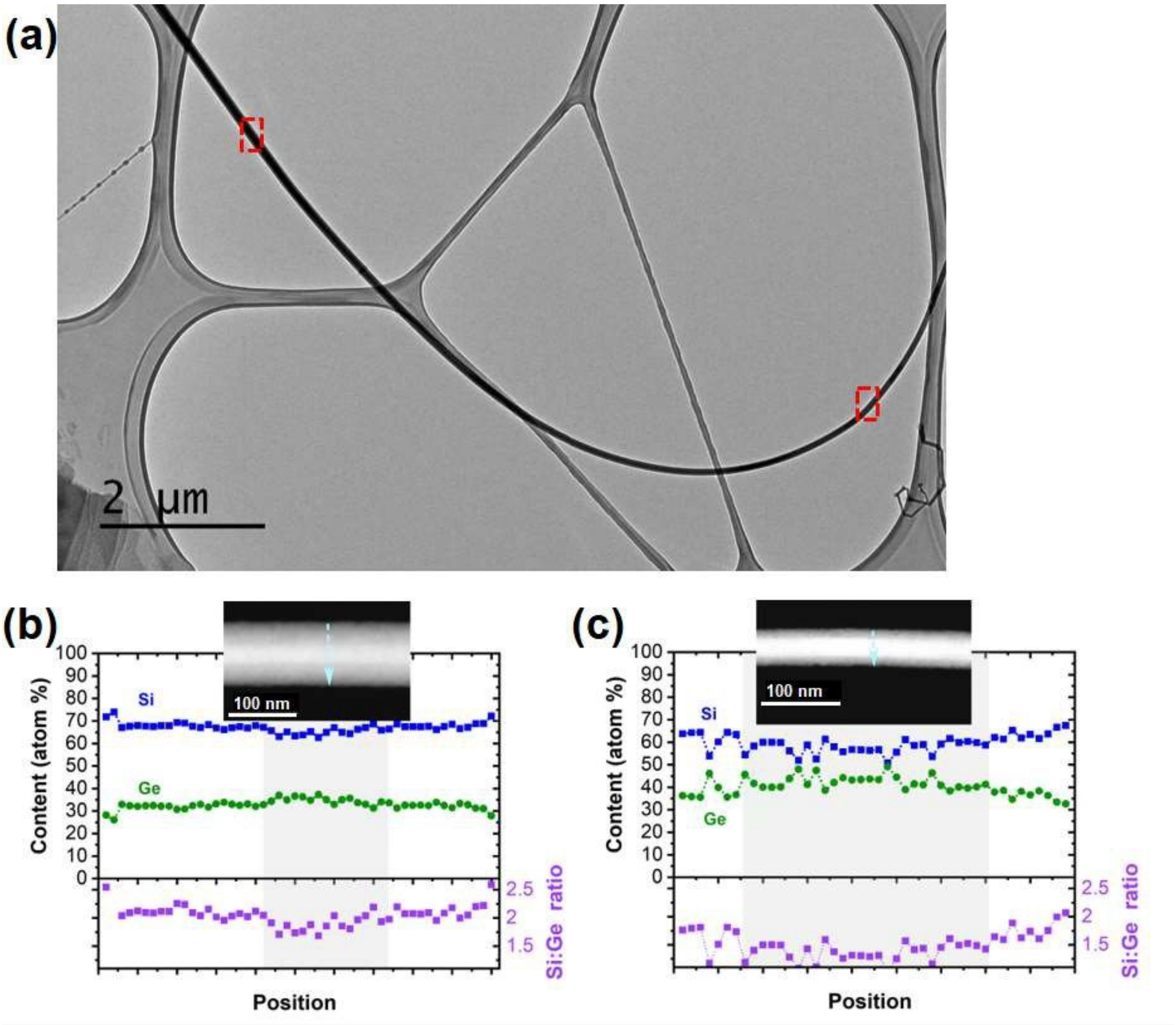

Figure S3. (a) TEM image of a $Si_{1-x}Ge_x$ NW grown at 783 K and marked regions used for recording

the cross-section EDX measurements. (b) Point EDX measurements perpendicular to the NW growth axis are performed along the arrows in the associated HAADF images for sections with (b) thicker and (c) thinner amorphous layer. The Si:Ge ratio shows the higher Si content close to 2 in the amorphous shell area. The crystalline core appears brighter in the HAADF and is illustrated by the grey background in the graphical illustration of the EDX content.

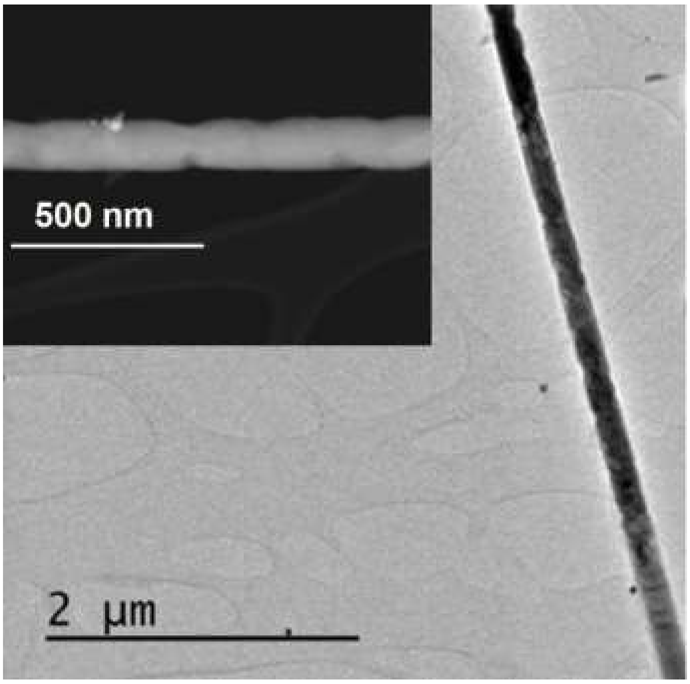

Figure S4. TEM image of a $Si_{1-x}Ge_x$ NW grown at 773 K, subsequent oxidation at 1073 K, and etching procedures using KI/I_2, followed by buffered HF shows a rough surface.
